# Health Care Provider Utilization and Cost of an mHealth Intervention in Vulnerable People Living With HIV in Vancouver, Canada: Prospective Study

**DOI:** 10.2196/mhealth.9493

**Published:** 2018-07-09

**Authors:** Amber R Campbell, Karen Kinvig, Hélène CF Côté, Richard T Lester, Annie Q Qiu, Evelyn J Maan, Ariane Alimenti, Neora Pick, Melanie CM Murray

**Affiliations:** ^1^ Division of Experimental Medicine Department of Medicine University of British Columbia Vancouver, BC Canada; ^2^ Oak Tree Clinic British Columbia Women's Hospital Vancouver, BC Canada; ^3^ Women's Health Research Institute British Columbia Women's Hospital Vancouver, BC Canada; ^4^ Department of Pathology and Laboratory Medicine University of British Columbia Vancouver, BC Canada; ^5^ Centre for Blood Research University of British Columbia Vancouver, BC Canada; ^6^ Division of Infectious Disease Department of Medicine University of British Columbia Vancouver, BC Canada; ^7^ Department of Pediatrics University of British Columbia Vancouver, BC Canada

**Keywords:** HIV, mHealth, health care provider, cost, health care utilization, adherence

## Abstract

**Background:**

Improving adherence to combined antiretroviral therapy (cART) can be challenging, especially among vulnerable populations living with HIV. Even where cART is available free of charge, social determinants of health act as barriers to optimal adherence rates. Patient-centered approaches exploiting mobile phone communications (mHealth) have been shown to improve adherence to cART and promote achievement of suppressed HIV plasma viral loads. However, data are scarce on the health care provider (HCP) time commitments and health care costs associated with such interventions. This knowledge is needed to inform policy and programmatic implementation.

**Objective:**

The purpose of this study was to approximate the resources required and to provide an estimate of the costs associated with running an mHealth intervention program to improve medication adherence in people living with HIV (PLWH).

**Methods:**

This prospective study of HCP utilization and costs was embedded within a repeated measures effectiveness study of the WelTel short-message service (SMS) mHealth program. The study included 85 vulnerable, nonadherent PLWH in Vancouver, Canada, and resulted in improved medication adherence and HIV plasma viral load among participants. Study participants were provided mobile phones with unlimited texting (where required) and received weekly bidirectional text messages to inquire on their status for one year. A clinic nurse triaged and managed participants' responses, immediately logging all patient interactions by topic, HCP involvement, and time dedicated to addressing issues raised by participants. Interaction costs were determined in Canadian dollars based on HCP type, median salary within our health authority, and their time utilized as part of the intervention.

**Results:**

Participant-identified problems within text responses included health-related, social, and logistical issues. Taken together, management of problems required a median of 43 minutes (interquartile range, IQR 17-99) of HCP time per participant per year, for a median yearly cost of Can $36.72 (IQR 15.50-81.60) per participant who responded with at least one problem. The clinic nurse who monitored the texts solved or managed 65% of these issues, and the remaining were referred to a variety of other HCPs. The total intervention costs, including mobile phones, plans, and staffing were a median Can $347.74/highly vulnerable participant per year for all participants or Can $383.18/highly vulnerable participant per year for those who responded with at least one problem.

**Conclusions:**

Bidirectional mHealth programs improve HIV care and treatment outcomes for PLWH. Knowledge about the HCP cost associated, here less than Can $50/year, provides stakeholders and decision makers with information relevant to determining the feasibility and sustainability of mHealth programs in a real-world setting.

**Trial Registration:**

ClinicalTrials.gov NCT02603536; https://clinicaltrials.gov/ct2/show/NCT02603536 (Archived by WebCite at http://www.webcitation.org/70IYqKUjV).

## Introduction

With the success of combined antiretroviral therapy (cART), the health of people living with HIV (PLWH) has greatly improved [1], and life expectancies are now approaching those of the general population [2]. However, cART effectiveness is dependent on high rates of medication adherence. Among vulnerable populations such as individuals at high risk for disengagement from treatment due to various social determinants of health (eg, housing or food insecurity, substance use issues, and advanced HIV infection), being adherent is often a challenge [3-5]. This can be due to a combination of demographic, structural, and psychosocial barriers, and it results in PLWHs’ engagement being lost along the cascade of care continuum [4,6]. Adherence is also crucial at the population level, as nonadherence can result in high HIV viremia, which places others at higher risk of acquiring the virus [7,8]. Consequently, tools to help cART adherence and engagement in care bring value for both personal and population health. It is known that patients’ improved connection to health care providers (HCPs) can enhance their engagement in care [9-13]. This can be achieved through the use of mobile health (mHealth) technology, whereby a mobile phone is used to connect a patient with an HCP [14-16]. When provided in a bidirectional fashion, mHealth provides a patient with the ability to request and receive assistance when not physically present at clinic. This allows a variety of “problems” related to physical, emotional and mental health, housing, food security, and medication use to be managed in timely fashion, as they arise [17,18]. In addition, mHealth enables HCPs to connect with patients and monitor their wellness between clinic visits [18].

Communication through mHealth technology with PLWH was first shown to significantly improve adherence to cART and plasma viral load (pVL) suppression in a text-messaging intervention study conducted in Kenya (WelTelKenya1) [14]. This bidirectional outpatient management service was then tested for acceptability and feasibility at Oak Tree Clinic, Vancouver, British Columbia, in a prospective mixed methods pilot study (WelTelBC1) [18,19]. HCPs involved with the pilot reported that, although workload increased initially, intervention benefits went beyond improving pVL and addressed the social determinants of health that act as barriers to engagement in care and to medication adherence [18,19]. This pilot was followed by the study presented here, WelTel OakTree, which examined the effectiveness of this weekly text-messaging intervention over one year, with 85 highly vulnerable and nonadherent PLWH, who experience many social determinants of health as barriers to care [20]. Before the study, communication with patients was likely to be only around their scheduled appointments, with the main method of communication being in person or perhaps over the phone on an intermittent basis. Efficacy results of WelTel OakTree demonstrated an improvement in cART adherence and HIV pVL among PLWH who received the intervention, so that 47.5% of participants achieved undetectable pVL by study end [20].

While the benefits of mHealth are clear, little is known regarding HCP time utilization and the costs involved in enacting such an intervention. Detailing the types of problems and time required by HCPs to provide the service would allow visualization for what is involved in providing this efficacious program, as a program that is very time intensive for providers may not be feasible or sustainable in busy clinics. Assessing the true financial and personnel costs to the health care system is crucial to the translation of this research into clinical care and is necessary information for health care system officials who may elect to provide such a service to their population. Prospectively and throughout the WelTel OakTree study [20], detailed data were collected on all HCP-participant interactions. Here we report the estimated HCP utilization and cost of providing an mHealth intervention for vulnerable PLWH in Canada.

## Methods

### Study Setting

The Oak Tree Clinic (OTC), located in Vancouver, Canada, is a provincial referral center for women and families living with HIV throughout British Columbia, many of whom have multiple demographic, structural, and psychosocial barriers to engagement in care. The OTC hosts an interdisciplinary team that provides holistic care for the health needs of women and their families in a single setting. The PLWH receiving care at OTC span all HIV-acquisition risk factor groups.

### Study Participants

Details of the WelTel OakTree study, its participants, and results were reported elsewhere [20]. Briefly, 85 participants were recruited between April 2013 and May 2014 at OTC and were enrolled in a repeated measures cohort study, with the 12 months prior to initiation of the study used retrospectively as the control year.

Patients were eligible for study participation if they met the following inclusion criteria: attendance at OTC for at least one year prior to study entry (with the one year prior to study start representing the control year), an indication for cART, detectable HIV pVL (≥200 copies/mL) in the control year, age ≥14 years, and being “vulnerable.” The latter was defined as being high-risk or vulnerable for disengagement from treatment according to a list of predetermined criteria. High-risk individuals were identified based on consensus by the care team where at least one of the following was present: intimate partner violence, unstable housing, advanced HIV infection/AIDS, mental health illness, cART nonadherence, difficulty to contact, poor appointment attendance, substance use, long distance from care, and recent incarceration. All candidates were reviewed by the multidisciplinary care team who decided on applicable vulnerabilities by manner of consensus. We excluded those not meeting the above criteria, living in an area with no mobile phone service, or otherwise unable to communicate via the text-messaging system.

Participants were provided with a basic mobile phone with unlimited text-messaging capability if they did not have one. Where required, participants received instruction on how to use text messaging for communication. In addition to the intervention, participants continued to receive their regular care through the interdisciplinary OTC team. This included follow-up appointments every 1-4 months, as clinically indicated by overall health status and HIV pVL. In British Columbia, cART for PLWH is fully covered through the provincial drug treatment program and was prescribed according to published provincial therapeutic guidelines [21]. The study was reviewed and approved by the University of British Columbia Research Ethics Board (H12-03002).

### WelTel Program

**Figure 1:**
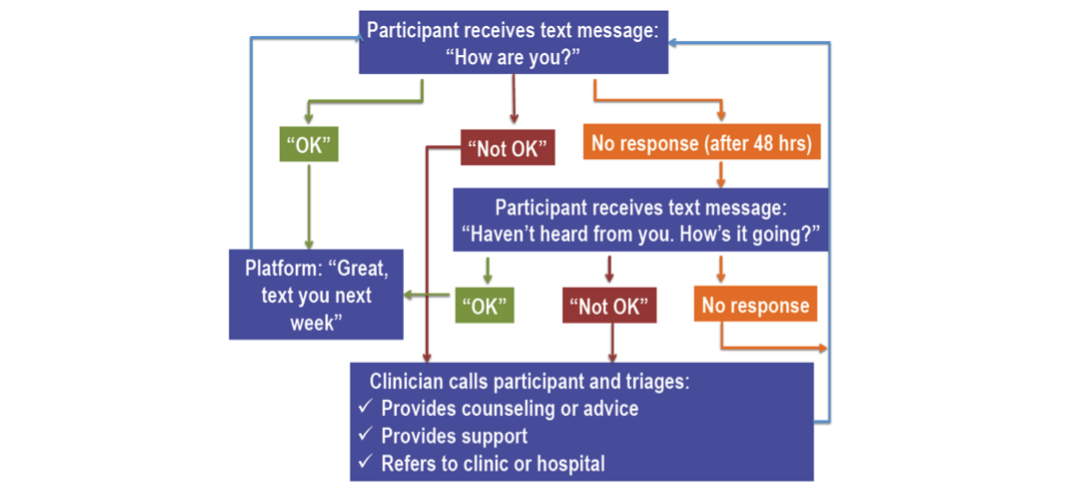
Weekly text-messaging structure.

Participants received a weekly interactive text message of “How are you?” to check in on their health status for one year. An automated software platform sent the text every Monday at noon from a number not traceable to the clinic. Participants were asked to respond each week within 48 hours if they were “OK” or had a “problem.” A study nurse monitored responses daily (except weekends) and responded to all “problem” texts from participants during working hours, as shown in Figure 1. The study nurse triaged “problem” responses and involved additional HCP as required. Participants who did not respond to the initial text were sent a second message on Wednesday, “Haven’t heard from you. How’s it going?” If there was still no response, the clinic nurse called participants who had not responded by Thursday. If there was again no response, participants were texted as per usual the following Monday. HCPs never texted information relating to HIV status or the clinic unless asked explicitly to do so by the participant.

### Data Collection and Analysis

All communications related to the program were recorded and encoded prospectively by participant ID in an electronic study log maintained exclusively by the single study nurse. The number of minutes required to triage and solve each “problem” response was also recorded in the log. At the time of the interaction, the study nurse thematically coded all problems based on the nature of each interaction and the classification of the HCP involved with the interaction. Where multiple providers were consulted, time taken and theme of interaction was recorded for each one. The cost of each interaction was then determined based on HCP time utilized and the mean salary of each HCP type, within our health authority (British Columbia’s Provincial Health Services Authority) [22]. Clinical data, including HIV pVL, CD4 (cluster of differentiation 4) counts, appointment attendance, and medication adherence, are reported elsewhere [20].

## Results

### Participant Characteristics

Of the 85 participants recruited, 80 remained enrolled for the entire duration of the study, and five withdrew for personal reasons. Baseline demographics of these 85 participants are shown in Table 1. Of the participants, 44 had their own mobile phone at study start, and 41 were given phones with unlimited text-messaging capability.

### Text-Messaging Responses

Over the intervention year, a total of 3764 “How are you?” texts were sent to participants, fewer than the predicted total of 4420 texts. Texts were not sent either due to planned participant absences (n=125), participants losing their phone (n=366), or participant withdrawal (n=165). At the participant level, a wide range of individual response rates to text messages were observed (0%-98%). The mean response rate was 50% (median 52%) among participants continuing to study completion. Of text messages sent, 46.68% (1757/3764) resulted in an initial “OK” response, 9.62% (362/3764) indicated a “problem,” and 43.70% (1645/3764) returned no response. While 362 “problem” responses were received in response to the Monday text message, an additional 203 “problem” responses were received later in the week, either as a second “problem,” or as a new problem after an initial “OK” response. Thus, at study completion, there were a total of 565 “problem” responses received.

**Figure 2:**
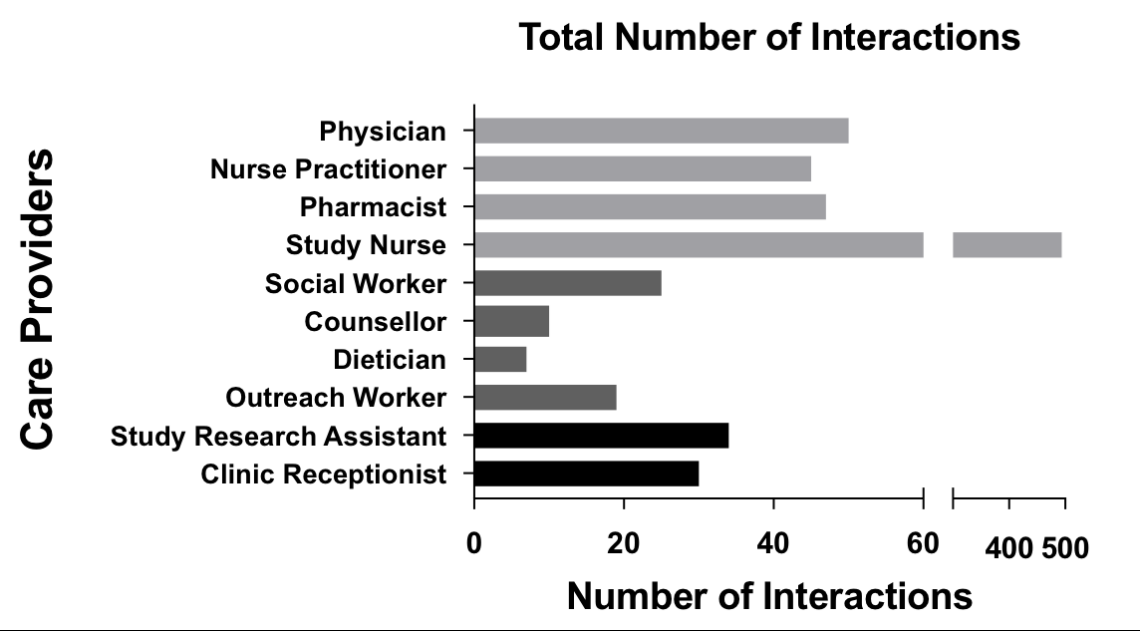
Total number of interactions with participants per health care provider over study year.

**Figure 3:**
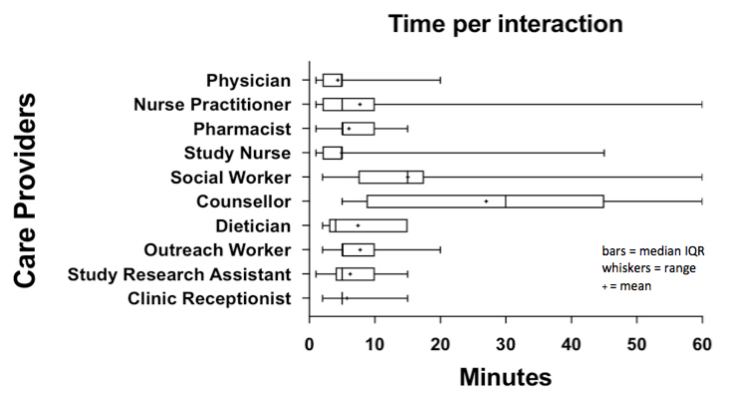
Total amount of health care provider time per interaction.

**Figure 4:**
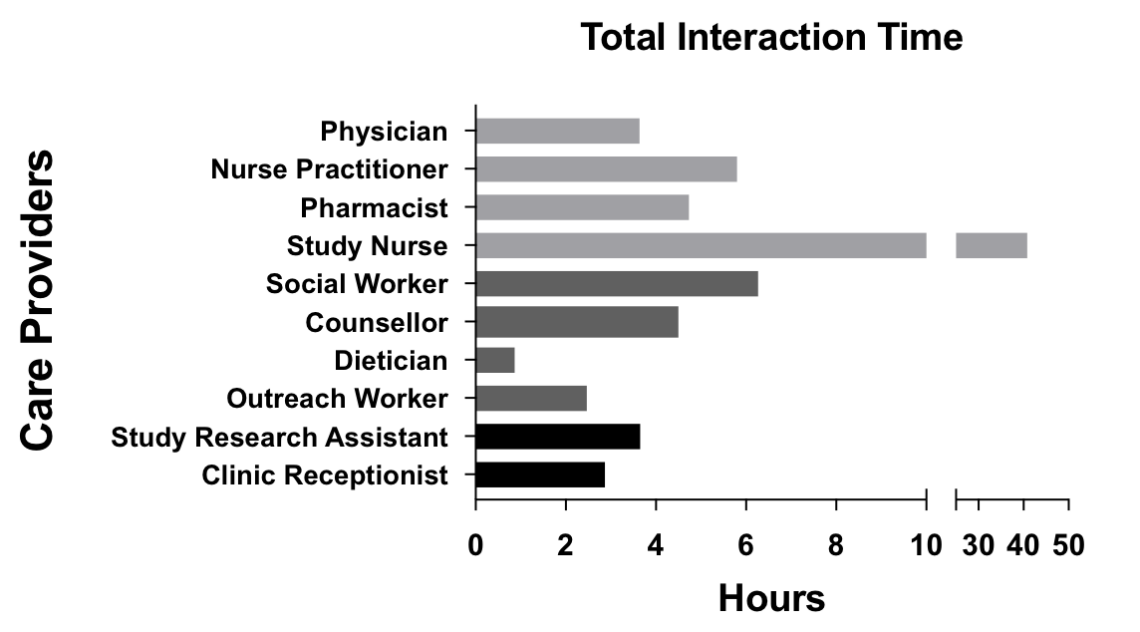
Total interaction time per health care provider over study year.

As more than one HCP was often required to address an issue, a total of 761 distinct HCP interactions resulted from the 565 problem responses. We illustrate the number of HCP-participant interactions by HCP type (Figure 2), mean time of each interaction (Figure 3), and total time per HCP (Figure 4) over the study period. The study nurse managed 64.9% (494/761) of problem interactions and spent the most time (2443/4533 minutes, 53.9%) on problem solving overall, among all HCPs. The counselor spent the greatest amount of time per interaction, averaging 27 minutes. Overall, managing “problem” responses required a total of 75.5 hours, with a median (interquartile range, IQR) of 43 (17-99) minutes of HCP time per year for participants who responded with problems (Table 2). Additionally, approximately 78 hours (55 minutes/participant/year) of HCP time was required for sending unscheduled text messages, weekday monitoring of the platform, and making phone calls to participants who did not respond to text messages.

**Figure 5:**
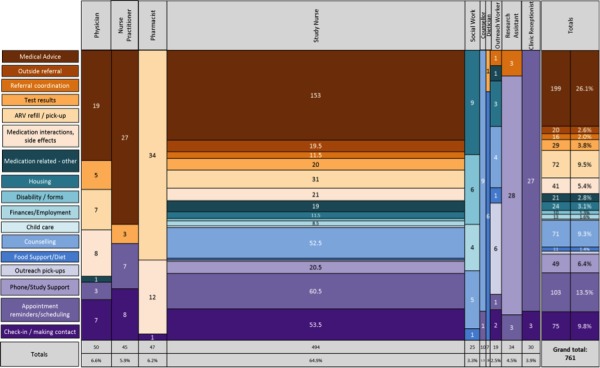
Nature and frequency of problem-response interactions that health care providers solved over intervention year.

WelTel participants reported a great breadth of “problems,” encompassing medical problems, as well as issues related to social determinants of health. Figure 5 depicts the nature and frequency of these “problem”-related interactions between HCPs and participants. The most common “problem” responses involved the participant seeking medical advice (199/761, 26.1%). The study nurse solved majority of these issues (153/199, 76.9%). Other common problem responses were related to counseling (71/761, 9.3%), antiretrovirals refill/pick-up (72/761, 9.5%), appointment reminders/rescheduling (103/761, 13.5%), check-in/making contact (75/761, 9.8%), and phone/study support (49/761, 6.4%).

**Table 1:** Baseline demographics of a high-risk Canadian HIV-positive cohort (N=80).

Demographics			Participants
**Gender, n (%)**			
	Female	72 (90)	
	Male	6 (8)	
	Transgender	2 (3)	
Age in years, median (range)			38 (15-61)
**Ethnicity, n (%)**			
	White	30 (38)	
	First Nations	27 (34)	
	African Canadian	18 (22)	
	South Asian	5 (6)	
**Income source, n (%)**			
	Disability	57 (71)	
	Welfare	6 (8)	
	Employed	4 (5)	
	Other	13 (16)	
**Cell phone ownership, n (%)**			
	Yes	43 (54)	
	No	37 (46)	

**Table 2:** Median time and cost per problem and per year for all participants and problem responders during WelTel intervention.

Times and costs		All participants (n=85)	Problem responders (n=65)
Median time/problem, minutes		5	5
**Time/year, minutes**			
	Median	22	43
	Interquartile range	3-85	17-99
	Range	0-335	2-335
Median cost/problem, Can $		4.08	4.08
**Cost/year, Can $**			
	Median	17.95	36.72
	Interquartile range	2.45-71.40	15.50-81.60
	Range	0-273.37	1.63-273.37

### Text-Messaging Costs

**Figure 6:**
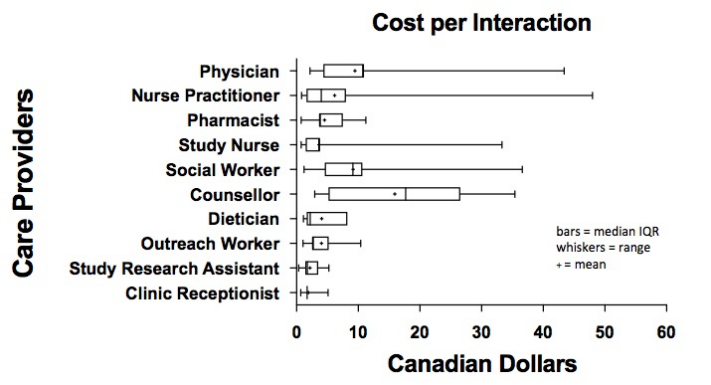
Cost per interaction per health care provider.

**Figure 7:**
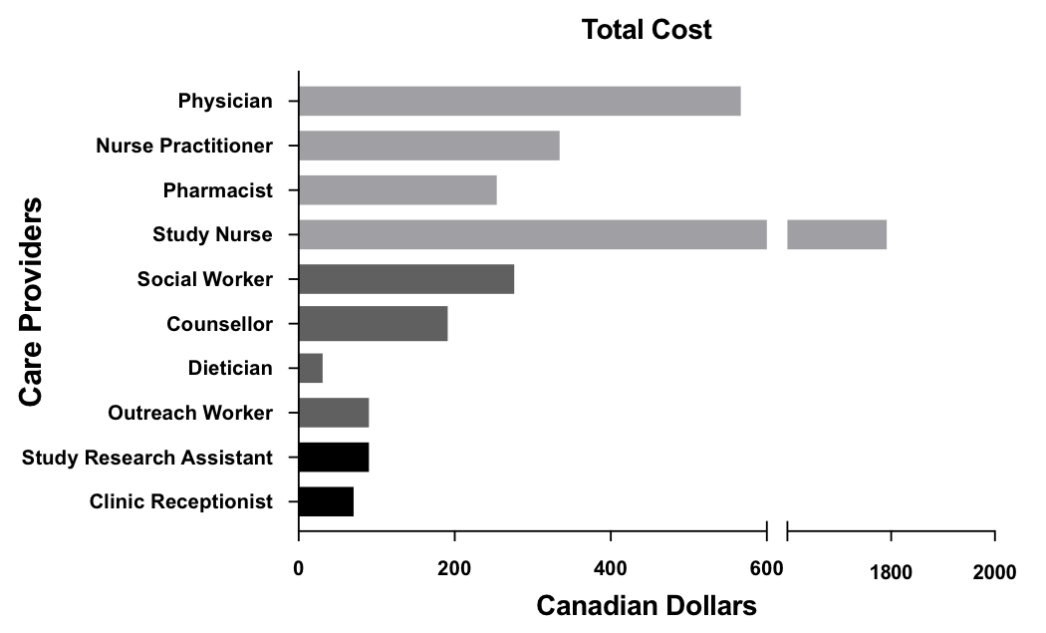
Total cost per health care provider over study year.

Figure 6 displays the cost per interaction and Figure 7 shows total costs (in Canadian dollars), over the study period for each HCP in managing “problem” responses. Interactions involving the counselor carried the greatest cost, averaging Can $19.11 per interaction, due to their length. However, these interactions were few (total cost Can $191.13). In contrast, interactions with the study nurse carried an average cost of Can $3.63 per interaction, for a total cost of Can $1791.70. The median cost of all HCP time for managing all “problem” responses in the study was Can $36.72 (IQR 15.50-81.60) per participant who had replied with “problem” responses, and the median cost of HCP time was Can $17.95 (IQR 2.45-71.40) per highly vulnerable participant for 1 year of service (Table 2).

The study nurse spent approximately 90 minutes each week monitoring participant responses, for a total cost of Can $3432.31 (including 20% benefits cost). The automated software platform cost for this study was Can $5000. Where a phone and phone plan were given to participants (n=50 over course of study), basic phone cost was Can $50, and cost of phone plans was Can $28.50 per person per month (including taxes). The total cost of providing phones and plans to 50 participants (of 85) was Can $392 per person per year, or a total cost of Can $19,600/year. Study cost per participant when a basic phone and unlimited texting plan were included was therefore Can $509.15 per person per year for all participants or Can $527.92 per person per year for problem responders. Study cost for participants who used their own phone was Can $117.15 per person per year for all participants or Can $135.92 per person per year for problem responders. Therefore, the intervention overall cost Can $347.74 per highly vulnerable participant per year for all participants or Can $383.18 per highly vulnerable participant per year for those who responded with at least one problem (Table 3).

At study end, nearly half (38/80, 47.5%) of participants had undetectable pVL (previously published [20]). Thus, WelTel program and HCP cost per undetectable pVL achieved was Can $835.03/year.

**Table 3:** Deconstructed costs of WelTel OakTree Study during intervention year (in Canadian dollars).

Item	Median cost per participant (n=85)	Median cost per problem responder (n=65)	Total cost (n=85)^a^
Automated software platform	$58.82	$58.82	$5000^b^
Study nurse checking responses	$40.38	$40.38	$3432.31
Managing “problem” responses	$17.95	$36.72	$3699.20
Phones (n=50)	$50.00	$50.00	$2500.00
Phone plans (n=50)	$342.00	$342.00	$17,100.00
Own phone, total	$117.15	$135.92	$31,731.20
Given phone and plan, total	$509.15^c^	$527.92^d^	$31,731.20
All	$347.74	$383.18	$31,731.20

^a^50 participants were provided with a phone.

^b^Fixed cost: does not change with enrollment and is subject to change.

^c^50 participants were provided with phones and plans.

^d^41 participants were provided with phones and plans.

## Discussion

### Principal Findings

Mobile health interventions, such as WelTel, are effective at improving cART adherence and pVL in PLWH [14,15,20]. However, translating this program from research to clinical care requires the buy-in and support of decision makers and payers. This study provides policy makers with the real-world cost and staff requirements to roll out such a program as a part of care for PWLH who are vulnerable to disengagement in care in a Canadian setting.

Data were captured at the time of each event; hence, results presented here precisely reflect the nature of “problems” and time utilization for each HCP-participant interaction in the study. The study nurse was able to address the majority (64.9%) of “problem” responses without referral to a secondary HCP. Many of these responses were medically related. Thus, when implementing bidirectional mHealth interventions in the real-world setting, it would be advantageous to employ HCPs capable of giving basic medical advice when answering text messages. Other “problems” were related to social determinants of health, reflecting the vulnerability of our study population. These were managed through a variety of OTC care providers. In the case of a limited resource setting, however, many of these issues could be managed by a skilled nurse, with the aid of a social worker in some instances. A situation with fewer care providers, though providing a less specialized service, may provide the added benefit of further strengthening patient-HCP relationships through increased interaction. This could be beneficial, as enhancing patient-provider relationships has been associated with improved adherence self-efficacy [23], as well as improvement in cART adherence [9,11], viral suppression [24], and overall health outcomes [25]. The possible downside of a small team, however, may include a larger effect on participants if and when a staffing change occurs.

The total HCP time for managing “problem” responses was 75.5 hours, or 43 minutes per participant per year. It should be noted that the highly vulnerable and complex nature of the study participants might have resulted in more problems, inflating HCP time above what may be expected in a more mixed cohort. An additional 78 hours (55 minutes per participant per year) was required for sending unscheduled text messages, weekday checking of the platform, and making phone calls to participants who did not respond to text messages. However, phoning nonresponders later in the week was time consuming with low yield and is not recommended going forward. Without these calls, considerable savings (30-60 minutes/week) could be achieved. Overall, the time spent by HCPs per participant over the study year was lower than anticipated, amounting to a cost similar to that of a single physician visit (Can $130/hour) [22]. This small time investment may be worthwhile, since mitigating problems as they arise can prevent progression of illness and decrease morbidity, thus reducing costs of health care over time [26,27].

Indeed, it is known that PLWH with sustained viral suppression have considerably lower non-cART direct medical costs [28]. As complications involving medication tolerance and adherence are solved, adherent individuals become healthier over time [1], requiring less frequent medical appointments and fewer hospital admissions [28,29]. These costs would add up, as the average cost of a hospital stay in British Columbia is approximately Can $6000 [30]. Furthermore, stable, virally suppressed individuals could use the WelTel program for viral-load-informed differentiated care, where text messaging would be sufficient to follow stable patients and allow less frequent patient-provider visits [31]. Thus, the WelTel intervention could potentially avoid some of these costs.

Through improved cART adherence and HIV viral suppression, the WelTel mHealth program can also be expected to lower risk of HIV transmission from participants to others, an important public health consideration [28,32-34]. The cost of treatment for individuals newly diagnosed with HIV in Canada is estimated at Can $250,000 over their lifetime [35]. When also including quality of life years (Can $380,000) and productivity loss (Can $670,000), the estimated lifetime cost of those newly diagnosed increases to Can $1.3 million [35]. Consequently, fewer HIV transmissions and therefore fewer new cases of HIV per year would result in a lower cost of HIV care [36]. These cost reductions would likely offset the cost of our mHealth intervention, making the cost of Can $835.03 per newly undetectable PLWH/year a seemingly worthwhile investment.

Importantly, the clinical and economical information from our intervention can be applied to other aspects of health care, such as diabetes care [37,38], eldercare [39], and many other chronic diseases where patient treatment fatigue is a known barrier [15,40-43]. As bidirectional mHealth offers patients real-time advice from HCPs and improves self-management of chronic diseases, the benefits may extend beyond what we are able to quantify. Implementing a triage system could be beneficial, such that the WelTel service is provided to our most vulnerable patients, but also more selectively to those most likely to use it and benefit from it, optimizing usage of health care funds. Mobile health is an accessible and practical method of communication, as the majority of Canadians have mobile phones. In our study, approximately half of participants had phones at baseline, speaking to the vulnerability of our cohort. This increased the costs of our service and would thus likely overestimate the costs of implementing mHealth programs into health care practices where a higher percentage of individuals had a mobile phone. Taken together, information on the costs and time required by HCPs to provide the WelTel service gives valuable insight into what implementation would mean in our and other settings.

### Limitations

Since HCP resource usage data were not collected during the control year, we cannot comment on any change in HCP resource utilization relative to the past or future if the program were extended. Identified participant vulnerabilities were not measured at study end, so potential change or improvement cannot be determined. In addition, this study was carried out by engaging the most vulnerable patients in our clinic; thus, resource use for a more stable population may be different than that presented here.

### Conclusion

To our knowledge, this is the first study detailing HCP time and resource utilization for an mHealth intervention in Canada. While further studies should address the question of change in resource utilization over time, both prior to and in the context of providing an mHealth intervention (eg, less time needed for social work/outreach, fewer clinical appointments), our study shows that weekly patient contact does not require a considerable amount of HCP time. When compared to the cost of a physician visit or hospital admission, it carries only a modest cost per participant for PLWH who are most vulnerable to morbidity and death.

## Abbreviations

cART: combined antiretroviral therapy
HCP: health care provider
HIV: human immunodeficiency virus
mHealth: mobile health
OTC: Oak Tree Clinic
PLWH: people living with HIV
pVL: plasma viral load
